# Mobile Phones: Vital Addiction or Lethal Addiction? Mobile Phone Usage Patterns and Assessment of Mobile Addiction among Undergraduate Medical Students in Telangana, India

**DOI:** 10.1155/2021/8750650

**Published:** 2021-10-20

**Authors:** Vinay Jahagirdar, Kaanthi Rama, Pranavi Soppari, M. Vijay Kumar

**Affiliations:** ^1^Department of Internal Medicine, University of Missouri Kansas City School of Medicine, Kansas City, MO, USA; ^2^Department of Community Medicine, Gandhi Medical College, Secunderabad, India

## Abstract

**Background:**

Increased mobile phone usage among undergraduate medical students causes a detrimental effect on their health. The main focus of this study is to determine the pattern of mobile phone usage among undergraduate medical students in Hyderabad, India, and the detrimental effect on their health due to excess mobile use.

**Materials and Methods:**

A cross-sectional study was conducted among undergraduate medical students from various medical colleges in Hyderabad, India, from September 2020 to January 2021. Data were collected from 626 respondents using a semistructured, pretested questionnaire. Smartphone Addiction Scale-Short Version (SAS-SV) was used to assess the risk of smartphone addiction. Microsoft Excel and SAS were employed to analyze the data. Associations were examined using Fisher's exact test.

**Results:**

100% of the respondents were using mobiles, with 83.2% spending more than 4 hours on them. Only 22% reported that no mobile use during classes. Half (51.6%) admitted to keeping their mobiles close by while sleeping. 84.3% used social networking apps via their mobiles. Common symptoms arising from prolonged mobile usage included eye strain (67.9%), blurred vision (31.4%), and numbness or tingling in palms (30.9%). 52.70% of the respondents were at high risk of mobile addiction according to SAS-SV. Screen time more than 4 hours was associated with high risk of mobile addiction (*p* < 0.0001). Significant association was found between high risk of mobile addiction and eye strain (*p* < 0.0001), blurry vision (*p*=0.0115), numbness/tingling in palms (*p* < 0.0001), and heat/tingling in the auditory area (*p* < 0.0001).

**Conclusion:**

The study shows the alarming rate of risk of smartphone addiction among medical students. Students can be encouraged to assess their mobile addiction status and become aware of the issue. More research may be performed to develop standardized tools for early identification of mobile addiction and appropriate therapies for its rectification.

## 1. Introduction

Mobile phones are electronic devices with a cellular radio system which can be used over a wide area, without a physical connection to a network. They have become ubiquitous in today's day and age, with easy Internet access making life convenient in terms of social networking, banking, shopping, or to seek knowledge. With the advent of several health-promoting apps, including those involving nutrition, fitness, and mindfulness, mobiles are being increasing used to promote a healthy lifestyle. India's mobile phone base is estimated to reach 820 million by 2022, placing India in the second position globally, in terms of population using mobile phones, after China [1, 2]. Mobiles are indispensable for medical students to be in touch with their peers and families as their curriculum entails long working hours where they are confined to the hospital. They are also used extensively as a tool for education and note taking, with increasing availability of e-books, medical podcasts, dosage calculators, and online lectures [3].

Symptoms such as headache, impaired concentration, and fatigue stemming from the prolonged mobile use have been documented [4]. Studies have shown that mobile usage can have a negative impact on biological systems, leading to increased stress, sleep disturbances, and depression [5, 6]. The World Health Organization has classified mobile phone addiction as a form of nonsubstance addiction [7]. Excessive use of mobiles is an acquired compulsive behavioural pattern, consuming time and resources. Despite of the awareness of the consequences, there is persistent escalation of this behaviour. New disorders such as nomophobia or “no mobile phone” phobia and ringxiety have emerged from the excessive use of mobiles. Nomophobia is a psychological condition where one has fear of being away from mobile phone connectivity [8]. It may manifest as anxiety, respiratory alterations, trembling, perspiration, agitation, disorientation, and tachycardia [8]. Ringxiety is a “phantom” experience where a person hallucinates that his mobile is ringing or vibrating, without actually receiving any call or message [9]. Authors have also described terms like textiety, textaphrenia, binge texting, and posttraumatic “text” disorder [10].

Prevalence of mobile addiction in Indian youth ranges between 39% and 44% [8]. Medical students are vulnerable to burnouts and emotion exhaustion. Coupled with high intensity academics, their stress can lead to mobile phone addiction [11]. Studies have shown that poor sleep quality in medical students is associated with over use of mobile phones, placing them at increased risk of various physical ailments and mental disorders [6]. The primary objective of this study is to determine the pattern of mobile phone usage among undergraduate medical students in Hyderabad, India. The secondary objectives include the assessment of the detrimental effect on health due to excess mobile use.

## 2. Materials and Methods

A cross-sectional, observational study was conducted between September 2020 and January 2021, among undergraduate medical (MBBS) students in Hyderabad, India.

The prevalence of smartphone addiction was expected to be 50%, as per a study conducted by Nikhita et al. [12]. A sample size of 384 was calculated with 95% confidence interval and a 5% margin of error. The final sample size was estimated to be 500 after considering nonrespondents.

A pretest was conducted among 20 students who were not included in the final study. Mobile phone usage patterns were evaluated using a self-administered questionnaire. After assessing the content validity, necessary modifications were made to the questionnaire.

The ensuing semistructured, anonymous questionnaire was shared with participants via Google Forms. Students were explained the reasons for conducting the study and informed consent was taken. No personally identifiable information was collected. Undergraduate medical students, ≥18 years of age, who were willing to participate in the survey were included. Those who did not give consent were excluded.

The questionnaire consisted of 38 compulsory questions, including those from the Smartphone Addiction Scale-Short version (SAS-SV) [13]. This scale is a 10-item, self-reported questionnaire, developed by the Korean team of Kwon et al. for assessment of smartphone addiction. It includes questions on mobile overuse, effect of mobile usage on concentration, health, relationships, and daily life. The effect of mobiles on these parameters were rated by the participants on the 6-point Likert scale, with options ranging from “strongly disagree” (1 point) to “strongly agree” (6 points). A score above 34 points indicates high risk of mobile addiction.

The questionnaire covered demographic details including age, sex, and year of the study in medical school. Psychographic variables included were attitude towards mobile usage and awareness regarding mobile dependence and associated anxiety. Questions related to the effect of mobiles on health, sleep, and physical fitness and the impact of the COVID-19 pandemic on the mobile use were put forward. At the end of the questionnaire, health education was imparted regarding the ill effects of mobile overuse and the ways to limit addicting behaviour.

Though expected sample size was 500, 626 responses were received. All responses were included. They were analyzed and computed on Microsoft Excel and SAS (university edition) to arrive at the results. Associations were evaluated using Fisher's exact test, with *p* value <0.05 taken as significant.

## 3. Results

**Figure 1:**
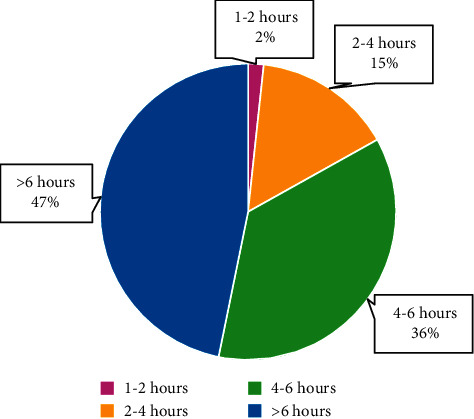
Mobile screen time among the respondents.

**Figure 2:**
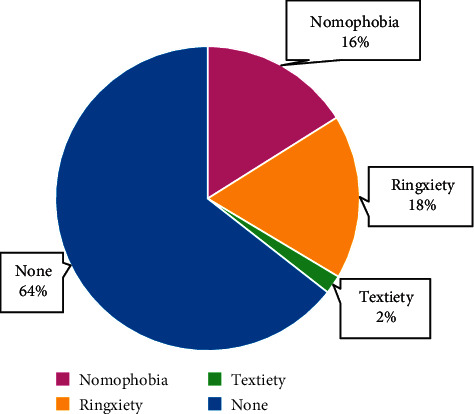
Awareness of terms related to mobile addiction.

The total number of respondents was 626. Mean age of the respondents was 20.14 ± 1.31 years, with majority of them (69.30%, *n* = 434) being females. All the respondents reported mobile usage (100%), with the mean age of getting their first mobile being 16.85 ± 1.94 years. 70% (*n* = 440) were using both mobile data and Wi-Fi to access Internet. Majority (83%, *n* = 521) were using their mobiles for more than 4 hours a day (Figure 1). Most of them (65%, *n* = 406) were not using a screen time monitoring app to measure usage. Almost 70% of the respondents (*n* = 432) were unaware of the terms nomophobia, ringxiety, and textiety (Figure 2). Close to 2/3^rd^ of the respondents (63.10%, *n* = 396) were using headphones, although only 11% (*n* = 68) were aware that headphone usage can reduce radiation exposure.

42% (*n* = 260) admitted to use mobiles during class for texting. Only 22% (*n* = 140) denied any mobile use during classes. Major mobile applications being used by students included those for social networking (84%), YouTube (74%), OTT streaming platforms (60%), and for online classes (48%). 90% (*n* = 565) reported an increase in screen time, since the onset of COVID-19 pandemic.

Kwon et al. suggested that males scoring above 31 and females scoring above 33 are at high risk of mobile addiction [13]. Females were given a higher cutoff since they were found to be more aware of their addiction and had higher self-reporting [13]. According to the suggested cutoffs by Kwon et al., 59.4% of the respondents were at “high risk” of mobile addiction (Table 1). Screen time of ≥4 hours per day was significantly associated with high risk of mobile addiction (*p* < 0.0001).

Responses to questions from the SAS-SV were noted (Table 2).

Close to 80% of the respondents (*n* = 497) did not switch off their mobile before sleeping, with most of the respondents (52%, *n* = 322) keeping it close by. A quarter (25%, *n* = 158) reported using their mobiles for an hour before sleeping. More than half (54%, *n* = 337) felt that their sleep cycle was affected due to mobile use.

4% (*n* = 26) admitted to using their mobiles while driving.

42% (*n* = 265) felt that mobile use restricted their day to day physical activities. Half (51.3%, *n* = 321) used physical fitness applications on their mobiles. 1 of 4 respondents avoided exercise to due increased mobile use (26%, *n* = 166)

Half of the respondents (51% *n* = 317) were aware that electromagnetic radiation from mobiles can cause health problems. 92% (*n* = 574) and 25% (*n* = 154) were aware that mobile usage can cause the detrimental effect on vision and diminished hearing, respectively. Side effects of prolonged mobile usage reported included eye strain (68%), heat/tingling in the auditory area (65%), blurred vision (31%), and numbness/tingling in palms (31%). Significant association was found between high risk of mobile addiction and eye strain (*p* < 0.0001), blurry vision (*p*=0.0115), numbness/tingling in palms (*p* < 0.0001), and heat/tingling in the auditory area (*p* < 0.0001) (Table 3).

## 4. Discussion

India has the fastest growing mobile user base in the world with 90 mobile connections per 100 of the population [14]. All the respondents in our study owned mobiles (100%), which is in concordance to other studies conducted among Indian medical students [15]. 59.4% were at high risk of mobile addiction according to the SAS-SV scale. This is similar to the burden estimated by other studies that utilized the SAS-SV scale—Dharmadikari et al. who found 46.15% addiction among 195 medical students in Maharashtra and Kumar et al. who found 44.7% addiction among 150 medical students pursuing internship in a tertiary care hospital in South India [16, 17]. However, it is higher than the 39–44% smartphone addiction rate reported in the general Indian population, in the meta-analysis and systemic review done by Davey and Davey [18]. This may be attributed to the long working hours medical students spend away from their families and friends, which make them more dependent on their mobiles.

81.1% reported more than 4 hours/day of screen time. This is substantially higher than the 60% reported by Ammati et al. and the 46% reported by Dasgupta et al. in 2017, among medical students from West Bengal [15, 19]. The ongoing COVID-19 pandemic, which has shifted teaching from lecture halls to online virtual classrooms, may be the cause for this discordance. Longer mobile screen time was significantly associated with increased mobile phone addiction (*p* < 0.0001). This is in concordance to the study published by Pavithra et al. among 200 medical students from Bangalore [20].

Mobile data were used to access the Internet by 93.80%, similar to the 87% reported by Basu et al. among 388 medical students from New Delhi in 2017 [21]. Cheaper access to mobile data has exponentially increased mobile data consumption in India [22].

Social media applications (84.4%) were more widely used than gaming apps (31.2%). This is similar to the results published by Tang et al. in 2017 who compared mobile addiction patterns amongst 3267 undergraduate students of various fields from the United States, China, and Singapore, which found that Asian students were using their mobiles more for networking, compared to their American counterparts, who used it more for gaming [23]. Ammati et al. also found that 46% students utilized their mobiles for social networking, compared to 7.9% who used it for gaming [15].

Only 22.40% reported that they did not use mobile phones during class hours. This is similar to the 15% reported by Siddiqi et al. in their study conducted among 129 medical students from Oman [24]. More than 3/4^th^ of the students use their mobiles during class hours, mainly for text messaging (41.50%). World-over, medical students are being encouraged to utilize electronic devices for educative purposes. However, misuse of these devices has become rampant. Students can be asked to switch off mobile phones during classes to minimize distractions.

Headphone usage was reported by 63.10%, a little lower than the 83% reported by Siddiqi et al. [24]. 43.7% were unaware of that headphone usage can help mitigate radiation risk from mobiles, whereas 90% of the Omanian students were aware of it [24]. More awareness needs to be spread regarding the use of hands-free devices or the speaker option, instead of keeping the mobile close to the ear. This may reduce the health risk caused by radio frequency radiations of mobile phones [25].

80% did not switch off their mobiles before going to bed, with 92% keeping them within 5 feet. This is similar to the results published by Siddiqi et al. where 70% left their mobiles on during bedtime [24]. More than half of our respondents (53.80%) felt that their sleep quality was affected due to mobiles. Studies have shown that increased mobile use is associated with reduced melatonin production and poor sleep quality [26, 27]. It is safer to turn off mobile data/Wi-Fi before sleeping and keep the mobile as far as possible. Shifting to a conventional bell alarm can help in keeping the mobile away.

65.7% agreed to have difficulty in concentration while working due to mobile use. This is substantially higher than the 26.4% reported by Basu et al. in 2018 among 388 medical students from New Delhi [21]. 62.9% of our respondents agreed that their friends and family complained that they use their mobile too much. This is way higher than the 20% reported by Aggarwal et al. in 2012 among 192 postgraduate medical students of a tertiary care institute in North India [28]. This shows that there is an increasing burden of the problems caused by mobile addiction over the years.

53% of our respondents believed that the mobile is a necessary tool to stay connected with their families, whereas 83% reported the same in a study conducted by Dixit et al. [29].

The strength of the present study is that its sample provides a good representation of medical students. It focusses on students, amongst whom mobile addiction is rampant. The main limitation of the study is that since it includes a relatively young and highly educated group, it cannot be generalized.

## 5. Conclusion

Mobile addiction has become a public health issue world-over. It has gained more limelight in developing countries like India, which have a younger population. The present study only shows the alarming increase in prevalence of smartphone addiction among young medical students. There is a pressing need for research into mobile phone usage patterns amongst medical students and doctors and the ill effects of the same. Students can be encouraged to assess their risk of mobile addiction status via online tools and become aware of this issue. Mobile addiction, like any other behavioural addiction, could be included in the Diagnostic and Statistical Manual of Mental Disorders (DSM-V**)**. More research studies can be done into standardized tools for early identification and appropriate therapies for rectification of mobile addiction.

## Figures and Tables

**Table 1:** Respondents with high risk of mobile addiction.

Gender (cutoff)	*N* (%)
Male (≥31)	123 (64.10%)
Female (≥33)	249 (57.40%)
Total	372 (59.42%)

**Table 2:** Responses to questions from the SAS-SV.

	Strongly disagree, *N* (%)	Disagree, *N* (%)	Weakly disagree, *N* (%)	Weakly agree, *N* (%)	Agree, *N* (%)	Strongly agree, *N* (%)
Missing planned work due to smartphone use	66 (10.5%)	98 (15.7%)	49 (7.8%)	136 (21.7%)	183 (29.2%)	94 (15%)
Having a hard time concentrating in class, while doing assignments, or while working due to smartphone use	46 (7.3%)	118 (18.8%)	51 (8.1%)	137 (21.9%)	172 (27.5%)	102 (16.3%)
Feeling pain in the wrists or at the back of the neck while using a smartphone	76 (12.1%)	133 (21.2%)	43 (6.9%)	149 (23.8%)	157 (25.1%)	68 (10.9%)
Would not be able to stand not having a smartphone	93 (14.9%)	155 (24.8%)	74 (11.8%)	105 (16.8%)	139 (22.2%)	60 (9.6%)
Feeling impatient and fretful when I am not holding my smartphone	132 (21.1%)	193 (30.8%)	87 (13.9%)	112 (17.9%)	68 (10.9%)	34 (5.4%)
Having my smartphone in my mind even when I am not using it	139 (22.2%)	219 (35%)	65 (10.4%)	89 (14.2%)	79 (12.6%)	35 (5.6%)
I will never give up using my smartphone even when my daily life is already greatly affected by it	164 (26.2%)	211 (33.7%)	84 (13.4%)	90 (14.4%)	57 (9.1%)	20 (3.2%)
Constantly checking my smartphone so as not to miss conversations between other people on Twitter or Facebook	162 (25.9%)	178 (28.4%)	53 (8.5%)	104 (16.6%)	101 (16.1%)	28 (4.5%)
Using my smartphone longer than I had intended	22 (3.5%)	60 (9.6%)	28 (4.5%)	111 (17.7%)	249 (39.8%)	156 (24.9%)
The people around me tell me that I use my smartphone too much	49 (7.8%)	126 (20.1%)	57 (9.1%)	128 (20.4%)	185 (29.6%)	81 (12.9%)

**Table 3:** Comparison of mobile usage patterns and symptoms arising from prolonged mobile usage patterns among those with high risk and those not at high risk of mobile addiction.

Variables	High risk of mobile addiction		*P* value^#^
	Present (*n* = 372), *N* (%)	Absent (*n* = 352), *N* (%)	
Gender			
Male	123 (19.6%)	69 (11%)	0.1335
Female	249 (39.8%)	185 (29.5%)	

Screen time			
<4 hours	33 (5.3%)	73 (11.7%)	<0.0001
≥4 hours	339 (54.1%)	181 (28.9%)	

Age			
≤20 years	251 (20.1%)	153 (24.4%)	0.07
>20 years	121 (19.3%)	101 (26.1%)	

Age of first mobile			
≤20 years	123 (19.6%)	65 (10.4%)	0.0508
>20 years	249 (39.8%)	189 (30.2%)	

Blurry vision			
Maybe	39 (6.2%)	19 (3%)	0.0115
Yes	129 (20.6%)	67 (10.7%)	
No	204 (32.6%)	168 (26.9%)	

Eye strain			
Maybe	45 (7.2%)	35 (5.6%)	<0.0001
Yes	281 (44.9%)	143 (22.8%)	
No	46 (7.3%)	76 (12.1%)	

Numbness/tingling in palms			
Maybe	45 (7.2%)	17 (2.8%)	<0.0001
Yes	135 (21.6%)	58 (9.3%)	
No	192 (30.7%)	179 (28.6%)	

Heat/tingling in the auditory area			
Maybe	42 (6.7%)	23 (3.7%)	<0.0001
Yes	117 (18.7%)	38 (6%)	
No	213 (34%)	193 (31%)	

^#^Fishers exact test.

## Data Availability

The literature review data used to support the findings of this study are included within the article.
